# Alginate-Based Edible Films Delivering Probiotic Bacteria to Sliced Ham Pretreated with High Pressure Processing

**DOI:** 10.3390/ijms18091867

**Published:** 2017-08-29

**Authors:** Foteini Pavli, Ioanna Kovaiou, Georgia Apostolakopoulou, Anastasia Kapetanakou, Panagiotis Skandamis, George-John E. Nychas, Chrysoula Tassou, Nikos Chorianopoulos

**Affiliations:** 1 Institute of Technology of Agricultural Products, Hellenic Agricultural Organization-DEMETER, Lycovrissi, Sof. Venizelou 1, 14123 Attica, Greece; photpavli@gmail.com (F.P.); ioannakovaiou@hotmail.com (I.K.); fns11007@fns.aegean.gr (G.A.); ctassou@nagref.gr (C.T.); 2 Laboratory of Food Microbiology and Biotechnology, Department of Food Science and Human Nutrition, Agricultural University of Athens, Iera Odos 75, 11855 Athens, Greece; gjn@aua.gr; 3 Laboratory of Food Quality Control and Hygiene, Department of Food Science and Human Nutrition, Agricultural University of Athens, Iera Odos 75, 11855 Athens, Greece; akapet@aua.gr (A.K.); pskan@aua.gr (P.S.)

**Keywords:** probiotic bacteria, high pressure processing, edible films, ham slices, pulsed field gel electrophoresis

## Abstract

The aim of the present work was to evaluate the efficacy of Na-alginate edible films as vehicles for delivering probiotic bacteria to sliced ham with or without pretreatment using high pressure processing (HPP). Three strains of probiotic bacteria were incorporated in Na-alginate forming solution. Ham slices (with or without pretreatment using HPP at 500 MPa for 2 min) were packed under vacuum in contact with the films and then stored at 4, 8 and 12 °C for 66, 47 and 40 days, respectively. Microbiological analysis was performed in parallel with pH and color measurements. Sensory characteristics were assessed, while the presence and the relative abundance of each probiotic strain during storage was evaluated using pulsed field gel electrophoresis. In ham slices without HPP treatment, probiotic bacteria were enumerated above 10^6^ CFU/g during storage at all temperatures. Same results were obtained in cases of HPP treated samples, but pH measurements showed differences with the latter ones exhibiting higher values. Sensory evaluation revealed that probiotic samples had a more acidic taste and odor than the control ones, however these characteristics were markedly compromised in samples treated with HPP. Overall, the results of the study are promising since probiotic bacteria were successfully delivered in the products by edible films regardless of the HPP treatment.

## 1. Introduction

During the last years, new trends are being observed in consumer demands regarding food products and diet habits. Interest is more focused on the active role of foods in well-being and life prolongation, as well as in their impact in the prevention of chronic diseases. As a result, a relatively new term “functional foods” is of great interest for both the industry and the consumers. Within the category of functional foods, probiotic supplemented foods obtain a remarkable position, with their market increasing annually [1]. Probiotic foods have been marketed mainly in the dairy and infant-food market, but the development of new non-dairy probiotic foods is considered essential. This is because lactose intolerance, cholesterol content, and allergenic milk proteins are the major drawbacks related to the intake of dairy products.

Additionally, consumers preferences in minimally processed foods have prompted researchers to focus on the application of innovative and alternative technologies for developing better quality products without compromising food safety [2]. High pressure processing (HPP) has a great potential in producing foods with an extended shelf-life by rendering food products microbiologically safer and hence ameliorating their quality. This emerging technology enables the reduction of spoilage microorganisms, while the population level of the surviving microbiota is keeping in low levels, during storage [3]. Recently, different HPP treatments have been applied in the food industry on various food products such as meat, fruits, fruit juices and vegetables. Advancements in the efficiency of HPP equipment has allowed this technology to be used in a wide variety of industrial applications with pressures ranging from 100 to 800 MPa depending on the objective [4]. With regards to the application of this technology on meat, HPP has been proven to promote lipid oxidation, volatile formation and induce color changes in sliced dry-cured ham [5,6]. Furthermore, HPP has an impact on the sensory characteristics of meat products [7]; however, limited research has been conducted to investigate the influence of HPP on the sensory properties of dry-cured ham [3].

Active packaging has been defined as a method of packaging in which the packaging material, the product and the environment interact during storage. Accordingly, the shelf-life is increased and the quality and safety of the products are improved [2]. Improvements in active packaging technologies have led to the development of bioactive food packaging systems that have the ability of presenting health benefits to the consumers. Bioactive agents can be incorporated into packaging materials, which can result in their gradual (and sometimes controlled) release to the food product. In the particular case of bioactive edible films and coatings, this release is not even required since the film or coating itself is supposed to be eaten with the food [8].

Due to the sensitivity of probiotics to common processing conditions such as heat treatment, acidic environment, high osmotic pressure and high redox potential, the design of an effective physicochemical barrier to stabilize the organisms is essential [9]. Such a possible solution was the incorporation of probiotic cultures into edible coatings, which was first proposed in 2007 by Tapia et al. [10] for application on fresh fruits. This concept may be expandable also to other surface contaminated foods, e.g., fresh meat and cooked meat products including frankfurters and ham slices. Since there are limited studies on this area, the development of films and coatings supplemented with probiotics still needs a lot of research.

In a previous study [11], we demonstrated that out of 47 strains that were screened for probiotic potential, 19 showed good behavior under simulated gastrointestinal conditions and were considered safe, thus possessing desirable in vitro probiotic properties. Based on the above, a subsequent challenge is the application of these strains in various foods, both for exploiting their probiotic properties and/or their antimicrobial effect for food preservation and food safety. The objectives of the present study were: (i) to develop a Na-alginate edible film based on the incorporation of probiotic cultures in the matrix; (ii) to examine the effectiveness of such films in probiotic delivery; (iii) to investigate the effect of HPP treatment; and (iv) to evaluate the effect of the probiotic cultures on the physicochemical and sensory characteristics of the ham slices.

## 2. Results

### 2.1. Microbiological Analysis

**Figure 1:**
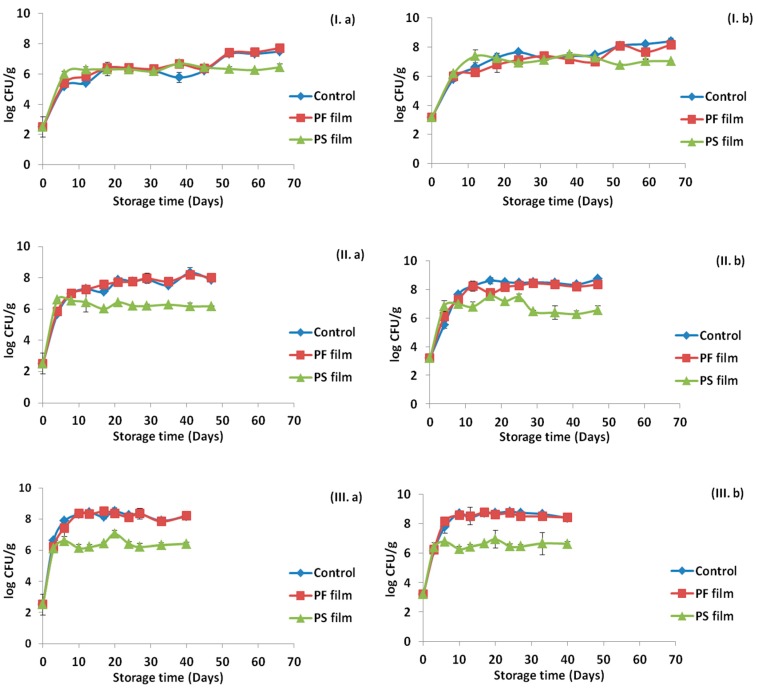
Changes in population levels of: (**a**) lactic acid bacteria; and (**b**) Total Viable Counts in ham slices with probiotic-supplemented edible films (PS) without high pressure processing (HPP) treatment during storage at: 4 °C (**I**); 8 °C (**II**); and 12 °C (**III**). Samples without edible films (controls) or with probiotic-free edible films (PF) were also studied.

**Figure 2:**
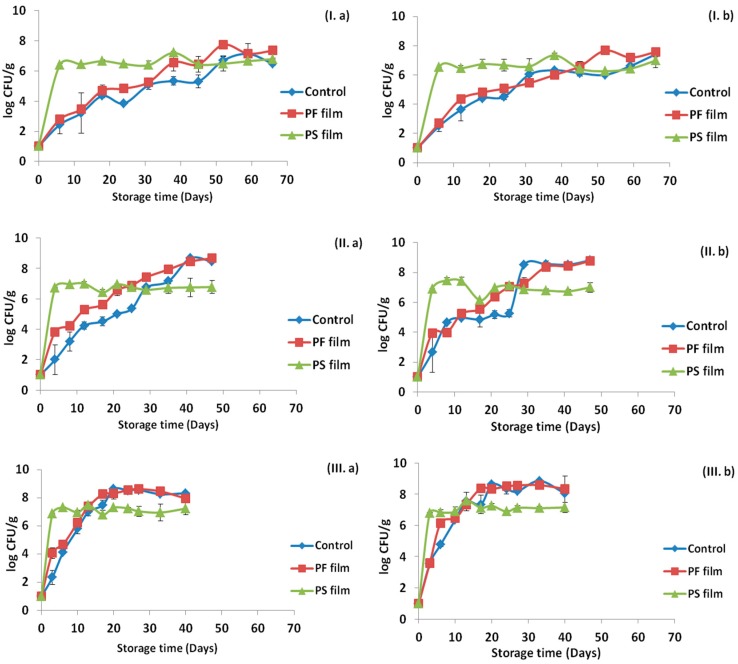
Changes in population levels of: (**a**) lactic acid bacteria; and (**b**) Total Viable Counts on ham slices with PS after HPP treatment during storage at: 4 °C (**I**); 8 °C (**II**); and 12 °C (**III**). Samples without edible films (controls) or with PF were also studied.

The initial population of the sliced ham was approximately 10^3^ CFU/g for both studied batches, which was relatively low and enabled the probiotic strains to be the dominant population in the ham. Lactic acid bacteria (LAB) and total viable counts (TVC) population levels for control samples, for samples in contact with probiotic-free edible films (PF) and for samples in contact with probiotic-supplemented edible films (PS) without prior HPP treatment for all storage temperatures, are presented in Figure 1. Regarding the samples with the probiotic-supplemented edible films, the LAB and TVC counts were maintained at levels of >10^6^ CFU/g in all temperatures during the storage period of the products. In Figure 2, changes in the population levels of LAB and TVC are presented for the samples treated with HPP during storage at 4, 8 and 12 °C. The storage temperature affected the LAB and TVC counts for all of the cases tested, and the differences were statistically significant (*p* < 0.05).

HPP treatment caused a reduction in the initial microbial population of the ham, therefore LAB and TVC were below detection levels in the beginning of the storage. After the first sampling for each storage temperature, LAB population in samples of probiotic-supplemented edible films was >10^6^ CFU/g and the levels remained constant during the shelf-life at 4, 8 and 12 °C. In the cases without HPP treatment, higher microbial counts were detected closer to the end of shelf-life (>10^8^ CFU/g) in control samples and samples with the probiotic-free edible films, whereas, for the HPP treated samples, the counts were always lower at the same time points. The pressure affected both the microbial populations, in all of cases apart for the case of 12 °C, and the differences were significant (*p* < 0.05). In every sampling point, other microbial populations were tested such as *Brochothrix thermosphacta*, *Pseudomonas* spp., Enterobacteriaceae, yeasts/molds and *Listeria monocytogenes*, but they were always below the detection limit.

### 2.2. Viability of the Incorporated Strains in the Na-Alginate Films

**Figure 3:**
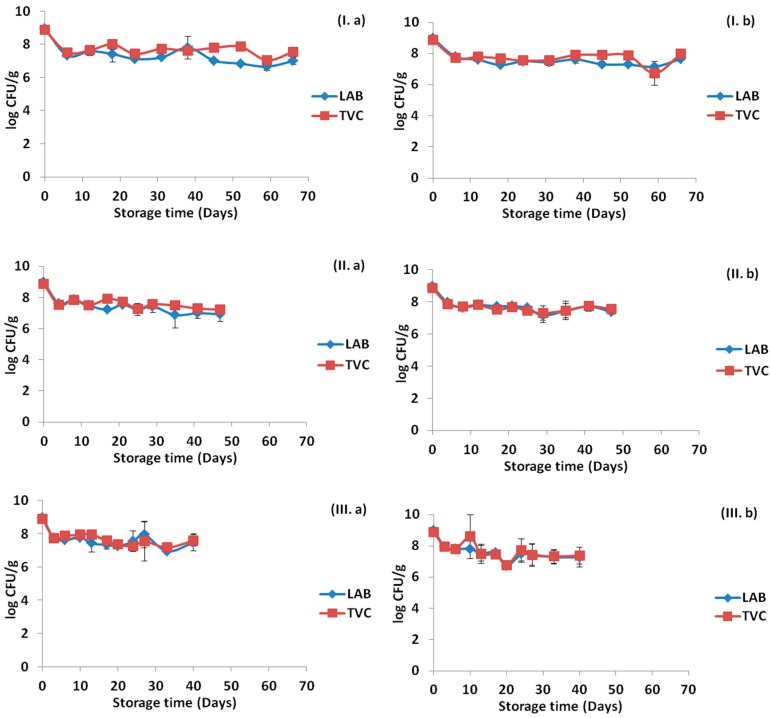
Changes in population levels of lactic acid bacteria (LAB) and total viable counts (TVC) in PS edible films in contact with ham slices: (**a**) without HPP treatment; and (**b**) after HPP treatment and during storage at: 4 °C (**I**); 8 °C (**II**); and 12 °C (**III**).

The viability of the incorporated strains was examined throughout the storage period in contact with the ham slices (66 days at 4 °C, 47 days at 8 °C and 40 days at 12 °C). Populations of LAB and TVC were determined for edible films stored at the three tested temperatures and the results are presented in Figure 3. As it can be seen, the storage temperature and the HPP treatment of the ham slices had no effect on the viability of the inoculated strains. In general, a reduction was detected in the films in the sampling point after their application in ham for all temperatures, however, it can be assumed that the drying process and subsequent stress had limited effect on the probiotic survival in adequate levels (>10^6^ CFU/g).

### 2.3. pH Determination

The pH results are presented in Table 1, Table 2 and Table 3. In the cases of the application of PS edible films, the pH values were affected and a rapid decrease was observed in all temperatures, regardless of the HPP treatment (*p* < 0.05). Overall, the application of HPP resulted in samples with higher pH values, while pH values were significantly (*p* < 0.05) affected by all factors (edible films, HPP, and storage time). The pH values recorded for samples with PS films are quite low for this type of ready-to-eat (RTE) ham and this was due to the high population levels of the probiotic bacteria.

### 2.4. Color Measurements

In Table 4, Table 5 and Table 6, the C* values are presented for each case and for the three temperatures. The application of edible films affected the mean values of C* and such differences were observed in all storage temperatures, however, only slightly. On the other hand, pressure did not affect the C* values in any case (*p* > 0.05). Previous studies suggest that HPP processing affects the C* value by causing an increase in L* value, a decrease in a* and no changes to b* values [12], although, in this study, similar results were not observed.

### 2.5. Sensory Evaluation

**Figure 4:**
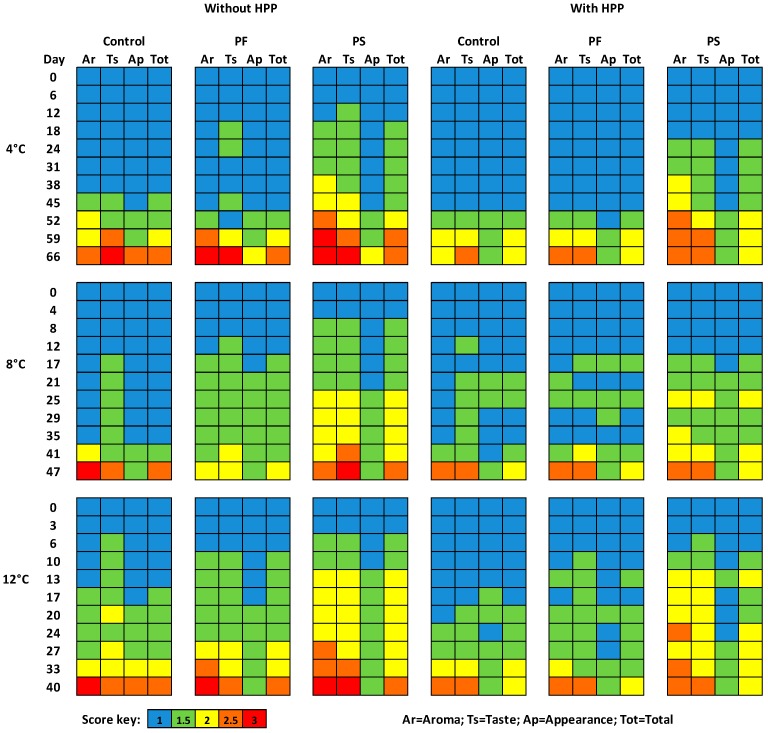
Sensory scores for ham slices treated or not with HPP, during storage at 4, 8 and 12 °C. The “Total” value represents the mean value of aroma, taste and appearance of each sample rounded to the closest value.

The results of the sensory assessment are presented in Figure 4. The presence of the PS film affected significantly the aroma and taste parameters and, as a result, the total scores in all of the temperatures tested. The HPP treatment, however, resulted in the production of less acidic samples with PS films and in better total score values from the panelists. Regarding the appearance scores, similar values were observed between pressurized and non-pressurized ham slices, while, at some time points, the appearance of the treated ones was evaluated as better. The period with ham showing acceptable sensory characteristics and overall appearance was considered typically as “shelf-life”. Total scores >2 indicated unacceptable quality. Since no major microbiological changes had occurred by that time, shelf-life was determined by only sensory evaluation. HPP application enhanced the sensory characteristics of the ham slices and lower scores (more fresh/less spoiled) were given from the panelists at the same time points compared to the untreated samples.

### 2.6. Probiotic Survival and Strain Differentiation in the Ham Slices and Edible Films

**Figure 5:**
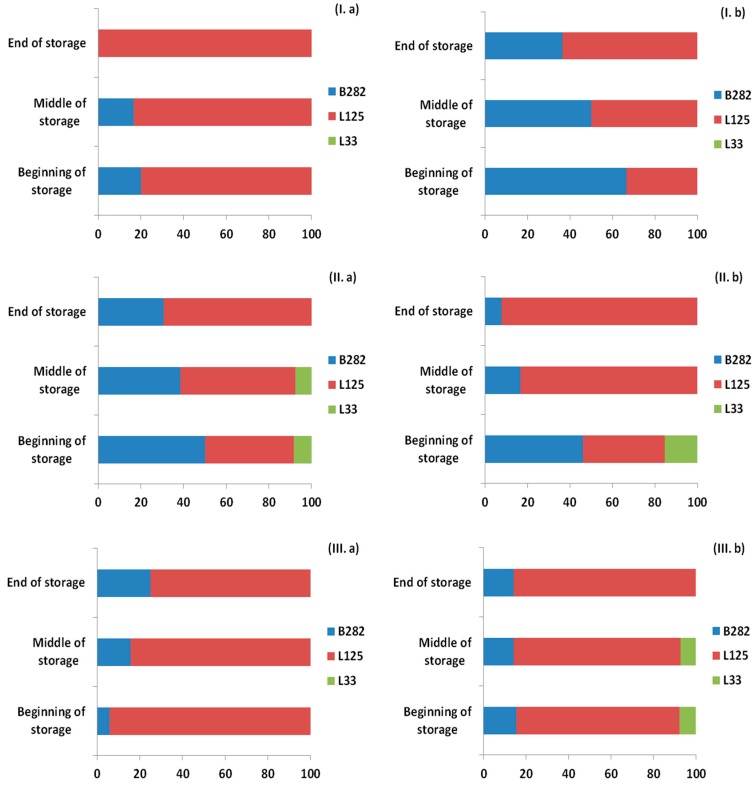
Distribution of isolates (%) of probiotic strains recovered from ham slices in three time points (beginning, middle and end) during storage at: 4 °C (**I**); 8 °C (**II**); and 12 °C (**III**); and (**a**) without HPP; or (**b**) with HPP treatment based on the pulsed field gel electrophoresis (PFGE) profiles.

**Figure 6:**
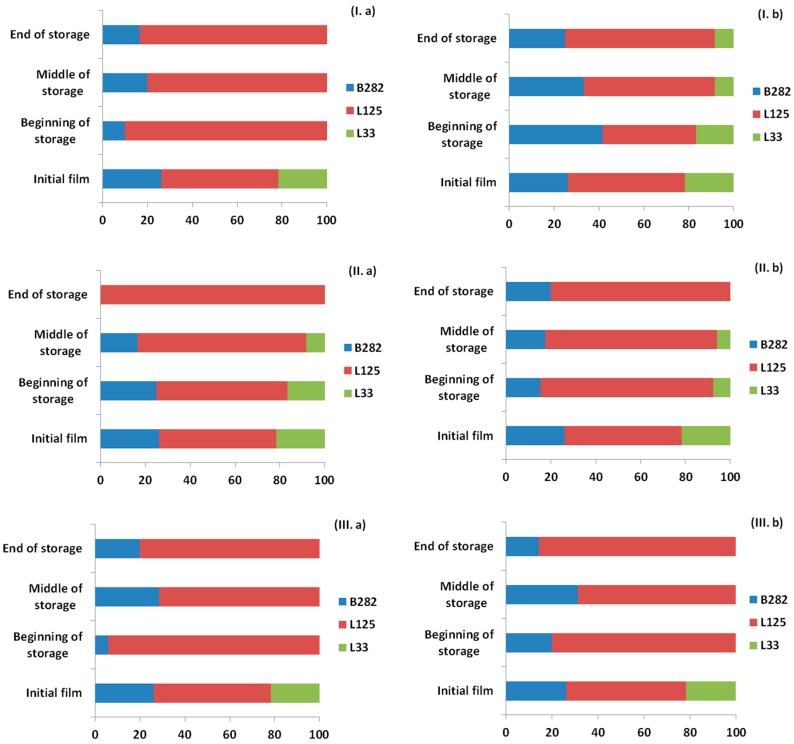
Distribution of isolates (%) of probiotic strains recovered films in contact with ham slices in three time points (beginning, middle and end) during storage at: 4 °C (**I)**; 8 °C (**II**); and 12 °C (**III**); and (**a**) without HPP; or (**b**) with HPP treatment based on the PFGE profiles.

As shown previously, Figure 1, Figure 2 and Figure 3 represent the LAB and TVC counts for the ham slices and the probiotic-supplemented edible films, respectively, during the storage at the three temperatures. Counts of LAB remained high (>10^6^ CFU/g) in both ham slices and films, therefore it was crucial to verify the presence of the film-incorporated probiotic strains. A total of 476 isolates (237 from ham samples and 239 from films) were recovered from petri dishes corresponding to the 6th or 7th dilution, and the presence of the incorporated strains was confirmed by pulsed field gel electrophoresis (PFGE). The results demonstrated that up to 100% of the microorganisms recovered, all belonged to the three probiotic strains that had been incorporated in the films with the distribution of each strain in each case to be presented in Figure 5 and Figure 6 for the ham slices and the edible films, respectively. The design of the experiment was such to have the same initial levels of the three strains in the ready-to-use edible films; however, this was not achieved, as presented in Figure 6. The initial distribution of the incorporated strains in the edible films before their direct application in ham slices were 26.1% for *Lb. plantarum* B282, 52.2% for *Lb. plantarum* L125 and 21.7% for *Lb. pentosus* L33. Results from the edible films revealed that *Lb. pentosus* L33 was present in the initial films prepared before their application, while it was not detected throughout the storage in cases of 4 °C without HPP treatment and 12 °C for both HPP, treated or not. Regarding the other cases, the aforementioned strain was detected in low percentages in the beginning and middle of storage time, whereas it was detected at the end of storage time, only in the case of HPP treatment. Higher percentages of presence in the films, observed for the strain *Lb. plantarum* L125 in all cases, a fact that was confirmed from the high percentage of this strain in the initial film concentration. With regards to the distribution of the strains in the ham slices, similar results were found with those obtained from the films. The strain *Lb. pentosus* L33 was detected in the beginning of storage in the cases of 8 °C (HPP or not) and in the case of the samples with or without pretreatment using HPP and stored at 12 °C, but with relatively low percentages, 15.4%, 8.3% and 7.7%, respectively. The strain *Lb. plantarum* L125 was the one with the higher percentage, similar to the results from the edible films and generally it is proven that the strain distribution for each case of the ham samples is similar to that of the edible films.

## 3. Discussion

Ham is among to the most popular RTE meat products and is mainly processed thermally during its production. HPP is an attractive preservation technology, and is relatively mild for meat products such as sliced ham, when low or moderate temperature and pressure combinations are applied. The efficacy of this technology has been reported previously for many different products including ham [13,14,15,16,17,18,19,20,21,22,23,24]. Findings of our study confirmed the hypothesis that HPP can be efficient in reducing the microbial populations in the pressure values tested (500 MPa for 2 min) in cooked ham slices.

Much research, on the other hand, has been conducted regarding novel packaging materials, especially biopolymer edible films, that can be applied in food products to increase the shelf-life or enhance food safety, by possessing antimicrobial substances [25,26,27,28,29]. Recent advances in this field include the incorporation of heat-sensitive bioactive materials, one such example being that of probiotic bacteria, although, to the best of our knowledge, there is scarcity of studies [9,30,31]. None of the latter studies examined the efficiency of the probiotic supplemented edible films on meat products, and thus the data obtained from this work are of major importance for the practical potential of applying probiotic cultures on cooked meat products.

The probiotic-supplemented films, were found to be efficient for probiotic delivery on the sliced ham, regardless its previous HPP treatment, in the desirable levels (>10^6^ CFU/g). This is crucial, since probiotic beneficial effects are dose dependent and the suggested daily intake ranges from 10^6^ to 10^9^ viable cells. Based on the capacity of producing biomass of probiotic cultures and the required amount of these cultures per product/unit/package, it is anticipated that the augmentation of the price of package is negligible. As such, we believe that the proposed technology is sustainable and cost effective for the food industry. From the microbiological results, it was observed that the major spoilage organisms of ham products were LAB, since no other microbial populations were detected during the experiment. Such results are justified due to the packaging under vacuum that was used. LAB in samples with PS films after the first sampling point exceeded the level 10^6^ CFU/g in all temperatures and their levels remained high until the end of shelf-life. It is notable that in contrary to non-treated samples (Figure 1), in HPP-treated samples, LAB population in PS films is lower than in PF, suggesting possible competitive effect of probiotic on natural lab. Together with the microbiological analyses of the ham samples, analysis was also performed to the PS films that were in contact with the ham to monitor the possible reduction in the viability of the incorporated probiotic bacteria. The obtained results were promising, since only 1–1.5 log reduction was observed, regardless the storage temperature. Such results are in agreement with those of a previous study [32], where the viability of *Lactobacillus sakei* remained almost unaffected when it was incorporated into sodium-caseinate edible films and stored at 4 and 25 °C for 30 days. Another study with similar results regarding the viability of the incorporated probiotic bacteria was that of Lopez de Lacey [29], in which the gelatin edible films were stored at 2 ± 1 °C and the viability of the tested *Bifidobacterium bifidum* and *Lactobacillus acidophilus* was fairly constant throughout storage time. However, to confirm the probiotic strains’ presence in both ham samples and edible films, PFGE was performed and strain distribution was assessed in specific time points for the three temperatures examined. All the isolates belonged to the incorporated probiotic strains, although analysis revealed that the performance of the inoculated probiotic strains in ham slices and edible films was strain specific. These results highlight that the selection of a probiotic strain to be incorporated, should be thoroughly tested in order to achieve its successful delivery to the food products.

Results from pH indicated that the ham samples with PS films had significantly (*p* < 0.05) lower values compared to the control ones and this can be connected with the high LAB population of these samples. When HPP was applied, the pH values of the aforementioned samples were low, but higher than before and this can be explained due to the changes that occur after the HPP process in meat samples. In a previous study of Souza et al. [12], higher values of pH in pork meat were observed after HPP with a difference of 0.46 compared to the control samples. It needs to be noted, however, that by the 2nd sampling day at all temperatures, PS samples had an unexpected low pH (<5.5) for cooked meat products, contrary to PF samples with the natural LAB present, without though having any negative sensory impact (Figure 4). Color values were determined in our study, since it is an important parameter that affects the evaluation of the ham quality by consumers. Color index was affected by the application of edible films (*p* < 0.05) and was slightly increased in all storage temperatures, whereas HPP treatment did not affect the color significantly (*p* > 0.05). Many parameters contribute to the final color of the HPP treated ham such as the fat and water content, the salt level, the applied values of pressure as well as the duration of the treatment. In other studies, HPP application, affected the color of the treated meat samples [33], while in another study no differences were mentioned [34].

Regarding their sensorial attributes, ham slices packed with PS edible films were evaluated as more acidic in aroma and taste than the control ones, as it can be assumed also from the pH values. Similar sensorial results were observed in the samples treated with HPP, but, in this case, they were characterized as less acidic. The intense acidification that occurred in these samples due to the inoculated probiotic strains is the major drawback of such films application. The appearance, on the other hand, was always evaluated with similar values to the control samples and regardless the HPP treatment. In general, HPP resulted in producing samples with better sensorial characteristics during the storage time, even for the temperatures of 8 and 12 °C in comparison with the untreated ones. Sensory acceptance of HPP treated meat products in general, depends on color, texture, aroma and taste modifications induced by the process. Problems of sensory acceptance occur with raw products, mainly because of visible color changes. Thermal processed or cured products such as ham are less modified by pressure [7]. Results obtained from the sensory assessment contribute to the better understanding of the effects of the different technologies studied in this work and the consumers should always consider such results since they are crucial for the future acceptability of the products.

## 4. Materials and Methods

### 4.1. Probiotic Strains and Ham Slices

Three strains of potentially probiotic bacteria were used in the present study: *Lactobacillus plantarum* B282, *Lactobacillus plantarum* L125 and *Lactobacillus pentosus* L33 which were previously isolated from table olives and meat products [11,35]. The pure cultures were stored at −80 °C in De Man-Rogosa and Sharpe broth (LabM, Lancashire, UK) supplemented with 20% (*v*/*v*) glycerol and the strains were subcultured twice before use. Commercial packages of ham slices were purchased from a local supermarket (Athens, Greece) (10 × 10 cm; 20 g). Three storage temperatures were used in the study (4, 8 and 12 °C), while two independent experiments were performed (ham slices produced by different manufacturer were used) and duplicate samples were studied in each experiment.

### 4.2. High Pressure Processing (HPP) Treatment

HPP treatment (when applied) was conducted at the pressure of 500 MPa for 2 min at room temperature (20 °C). Pressure and temperature were constantly monitored and recorded (in 1 s intervals) during the process. Pressurization time reported does not include the pressure come-up and release times. Further details of the high pressure system and operating conditions can be found in previous papers [36,37].

### 4.3. Preparation of Na-Alginate Edible Films

The preparation of Na-alginate edible films was conducted as reported previously by Kapetanakou et al. [2]. Briefly, quantity of 2 g of Na-alginate (Applichem GmbH, Darmstadt, Germany) was added gradually in 100 mL of pre-warmed (65 °C) distilled sterile water and under stable agitation for complete dissolution. One mL of glycerol (plasticizer) was added in order to improve film flexibility and the forming solution was kept at 4 °C for 30 min to lower the temperature, until the addition of the probiotic cultures. A mix of the three probiotics was added with agitation, in a final population of 10^9^ CFU/mL in the forming solution (probiotic-supplemented edible films-PS). Na-alginate solution without the addition of probiotic cultures was also prepared (probiotic-free edible films-PF). Films were produced in different Petri-dishes using 20 g of Na-alginate solution and then were placed in a laminar flow cabinet to dry at ambient temperature for 12 h. Following drying process, aliquots of 20 mL of 2% *w*/*v* CaCl_2_ were added for 1 min, in order to detach the square films (ca. 0.5 g) from the Petri-dishes.

### 4.4. Microbiological Analysis

Samples (10 g) of ham slices were weighed aseptically, added to sterile quarter strength Ringer’s solution (LabM, Lancashire, UK) (90 mL), and homogenized in a stomacher (Stomacher 400, Circulator, Seward) for 60 s at room temperature. The resulting suspensions were serially diluted in the same diluent and 1 or 0.1 mL samples of the appropriate dilutions were poured or spread, respectively, on the following agar media: de Man–Rogosa–Sharp Agar (MRS, Oxoid, Hampshire, UK) for LAB, incubated at 30 °C for 72 h; Plate Count Agar (LabM, Lancashire, UK) for TVC, incubated at 30 °C for 48 h; STAA Agar Base (Oxoid, Hampshire, UK) for *Brochothrix thermosphacta*, incubated at 25 °C for 48 h; Rose Bengal Chloramphenicol Agar (LabM, Lancashire, UK) for yeasts/molds incubated at 25 °C for 5 days; Violet Red Bile Glucose Agar (Oxoid, Hampshire, UK) for Enterobacteriaceae, incubated at 37 °C for 24 h, Pseudomonas Agar Base (LabM, Lancashire, UK), for *Pseudomonas* spp. incubated at 25 °C for 48 h, as well as Palcam Agar Base (LabM, Lancashire, UK), for *Listeria* spp. incubated at 30 °C for 48 h.

### 4.5. Viability of the Probiotic Strains Incorporated within the Film

The viability of the incorporated strains was tested in films that were in contact with the ham samples at the same time intervals with the ham samples. The films were removed aseptically from the slices and placed in a sterile stomacher bag and homogenized for 120 s. Decimal dilutions were prepared in the same medium and 1 mL of the appropriate dilutions were poured on MRS agar and incubated at 30 °C for 72 h.

### 4.6. pH Values

The pH value of the samples was measured with a digital pH meter (HI 2211 pH-ORP Meter, HANNA Instruments, Woonsocket, RI, USA). The pH of the ham slices was measured in the ham homogenate (stomacher homogenate) after the end of the microbiological analysis.

### 4.7. Color Measurements

The ham color was assessed by taking at least 5 random measurements from the surface of the different ham samples using a Minolta Chroma Meter fitted with a CR-300 measuring head (Minolta, Osaka, Japan). The CIE (Commission Internationale de l’Eclairage) L*, a*, b*, colorimetry system was used for color determination with L* indicating lightness, a* indicating redness and b* indicating yellowness. Measurements of the instrument were standardized with respect to a white calibration plate. Color measurements avoiding the area with excess fat were taken and the values were recorded in order to determine C* (chroma).

### 4.8. Sensory Evaluation

Sensory evaluation of ham slices was performed during storage in all temperatures according to Gill and Jeremiah [38] by a sensory panel composed of five members (staff from the laboratory) at the same time intervals with microbiological sampling points. The same trained personnel were used in each evaluation and were all blinded to the sample tested. The evaluation was carried out under artificial light at ambient room temperature. The descriptors selected were based on the perception of aroma, taste and appearance. Each attribute was scored on a three-point hedonic scale ranging from 1 (fresh) to 3 (unacceptable). We used this scale because our aim was to evaluate the changes in aroma, taste and appearance regarding the spoilage, and not the preference. The same hedonic scale and with a panel of 5 members was used in many other studies aiming to evaluate spoilage status or shelf-life of meat products such as minced beef and pork [39,40]. Intermediate sensory qualities were attributed to scores of 1.5, 2 and 2.5. Specifically, a score of 1.5 was characterized deteriorated and was the first indication of change from that of typical fresh ham (i.e., less vivid color, aroma and taste slightly changed, but still acceptable by the consumer). Scores >2 characterized the product spoiled and indicated the end of shelf-life.

### 4.9. Pulsed Field Gel Electrophoresis (PFGE) for Monitoring Probiotic Survival and Strain Differentiation

In total, 476 isolates (237 from ham samples and 239 from films) were recovered from the highest dilution in MRS agar and were then screened with PFGE to determine the survival of the inoculated probiotic strains in levels ≥10^6^ CFU/g and the differentiation during the storage period at the three temperatures tested without or after the HPP treatment. In brief, genomic DNA extraction was performed from all isolates, as previously reported [41]. The restriction enzyme *Sma*I (10U) (New England Biolabs, Ipswich, MA, USA) was used according to manufacturer recommendations for 16 h. Following digestion, restriction fragments were separated in 1% PFGE grade agarose gel in 0.5 mM Tris-Borate buffer on a CHEF-DRIII (BIO-RAD, Hercules, CA, USA) equipment with the following running parameters: 6 V/cm, 1 s initial switching time, 10 s final switching time and 16 h total run at 14 °C. Gels were then stained with ethidium bromide (Sigma-Aldrich, Schnelldorf, Germany) (0.5 mg/L) in water for 1 h and distained for 2 h before being photographed with GelDoc system. Conversion, normalization and further analysis were performed using the Pearson coefficient and UPGMA cluster analysis with Bionumerics software, version 6.1 (Applied Maths, Sint-Martens-Latem, Belgium).

### 4.10. Statistical Analysis

All experiments were carried out in duplicate with two independent batches of ham slices each. Analysis of variance was performed and means were separated with Duncan’s multiple range test. Significance was established at p < 0.05. The differences of the dependent variables regarding the factor “Pressure” were evaluated using the Student T-test in order to compare the means of the two different groups (with and without HPP treatment) to verify statistically significant differences between them. The statistical analysis was conducted using IBM^®^ SPSS^®^ Statistics for Windows software, Version 24.0 (IBM Corp., New York, NY, USA).

## 5. Conclusions

The application of sodium-alginate edible films supplemented with probiotic bacteria as a vehicle for delivery in RTE ham slices was found to be successful. The new products of ham had different organoleptic characteristics from the control samples, with the major difference being their acidic aroma and taste, while the appearance remained the same. HPP application was efficient in reducing the microbial population levels prior to edible film application and it did not affect negatively the sensory attributes of the products. Furthermore, HPP increased the shelf-life of the ham slices in all cases tested, exhibiting at the same time points better organoleptical attributes.

## Figures and Tables

**Table 1:** Changes in pH values for ham slices treated or not with HPP, during storage at 4 °C.

pH Values at 4°C						
Days	Without HPP Treatment			With HPP Treatment		
	Control	PF Film	PS Film	Control	PF Film	PS Film
**0**	6.49 ^A,a^ ± 0.07	6.49 ^A,a^ ± 0.07	6.49 ^A,a^ ± 0.07	6.54 ^A,a^ ± 0.11	6.54 ^A,a^ ± 0.11	6.54 ^A,a^ ± 0.11
**6**	6.53 ^A,a^ ± 0.06	6.24 ^B,a^ ± 0.01	5.22 ^B,b^ ± 0.23	6.52 ^A,a^ ± 0.00	6.30 ^B,b^ ± 0.01	5.43 ^B,c^ ± 0.07
**12**	6.35 ^B,a^ ± 0.03	6.11 ^C,b^ ± 0.01	4.77 ^C,c^ ± 0.04	6.45 ^A,B,a^ ± 0.01	6.30 ^B,b^ ± 0.01	5.23 ^C,c^ ± 0.01
**18**	5.76 ^C,b^ ± 0.01	6.04 ^C,a^ ± 0.02	4.44 ^D,c^ ± 0.01	6.42 ^A,B,a^ ± 0.03	6.31 ^B,b^ ± 0.01	5.05 ^D,c^ ± 0.01
**24**	5.54 ^D,b^ ± 0.03	5.66 ^D,a^ ± 0.01	4.32 ^D,E,c^ ± 0.02	6.40 ^A,B,a^ ± 0.00	6.14 ^C,b^ ± 0.01	4.91 ^E,F,c^ ± 0.01
**31**	5.55 ^D,a^ ± 0.05	5.44 ^E,a^ ± 0.04	4.36 ^D,E,b^ ± 0.01	6.48 ^A,B,a^ ± 0.01	5.72 ^D,b^ ± 0.02	4.77 ^G,c^ ± 0.03
**38**	5.52 ^D,a^ ± 0.04	5.44 ^E,b^ ± 0.00	4.40 ^D,E,c^ ± 0.01	5.76 ^D,a^ ± 0.07	5.75 ^D,a^ ± 0.01	4.93 ^E,b^ ± 0.00
**45**	5.52 ^D,a^ ± 0.01	5.44 ^E,a^ ± 0.07	4.36 ^D,E,b^ ± 0.01	6.01 ^C,a^ ± 0.01	5.43 ^E,b^ ± 0.07	4.84 ^F,G,c^ ± 0.03
**52**	5.39 ^E,a^ ± 0.01	5.25 ^F,a^ ± 0.16	4.22 ^E,b^ ± 0.01	5.83 ^D,a^ ± 0.04	5.26 ^G,b^ ± 0.02	4.78 ^G,c^ ± 0.01
**59**	5.27 ^F,a^ ± 0.03	5.17 ^F,b^ ± 0.01	4.26 ^E,c^ ± 0.01	5.73 ^D,a^ ± 0.11	5.33 ^F,b^ ± 0.07	4.68 ^H,c^ ± 0.04
**66**	5.36 ^E,a^ ± 0.01	5.25 ^F,b^ ± 0.01	4.25 ^E,c^ ± 0.03	5.62 ^E,a^ ± 0.03	5.40 ^E,F,b^ ± 0.07	4.66 ^H,c^ ± 0.04

^A,B,C,D,E,F,G,H^ Means with different uppercase letters within the same treatment are significantly different (*p* < 0.05). ^a,b,c^ Means with different lowercase letters within the same storage day are significantly different (*p* < 0.05).

**Table 2:** Changes in pH values for ham slices treated or not with HPP, during storage at 8 °C.

pH Values at 8 °C						
Days	Without HPP Treatment			With HPP Treatment		
	Control	PF Film	PS Film	Control	PF Film	PS Film
**0**	6.49 ^A,a^ ± 0.07	6.49 ^A,a^ ± 0.07	6.49 ^A,a^ ± 0.07	6.54 ^A,a^ ± 0.11	6.54 ^A,a^ ± 0.11	6.54 ^A,a^ ± 0.11
**4**	6.35 ^B,a^ ± 0.06	6.36 ^B,a^ ± 0.00	4.87 ^B,b^ ± 0.02	6.52 ^A,a^ ± 0.01	6.26 ^B,b^ ± 0.01	5.40 ^B,c^ ± 0.03
**8**	5.49 ^C,D,a^ ± 0.01	5.46 ^C,a^ ± 0.05	4.52 ^C,b^ ± 0.00	6.51 ^A,a^ ± 0.02	6.32 ^B,b^ ± 0.01	4.98 ^C,c^ ± 0.00
**12**	5.56 ^C,a^ ± 0.02	5.37 ^D,b^ ± 0.04	4.33 ^D,c^ ± 0.04	6.52 ^A,a^ ± 0.01	6.04 ^C,b^ ± 0.04	4.72 ^D,c^ ± 0.03
**17**	5.47 ^C,D,a^ ± 0.04	5.25 ^E,b^ ± 0.01	4.37 ^D,c^ ± 0.01	6.50 ^A,a^ ± 0.07	5.96 ^D,b^ ± 0.04	4.66 ^E,c^ ± 0.01
**21**	5.45 ^D,E,a^ ± 0.06	5.19 ^E,F,b^ ± 0.01	4.27 ^E,c^ ± 0.03	6.26 ^B.a^ ± 0.06	5.92 ^D,E,b^ ± 0.01	4.63 ^E,F,c^ ± 0.04
**25**	5.43 ^D,E,F,a^ ± 0.04	5.23 ^E,F,b^ ± 0.01	4.14 ^H,c^ ± 0.03	6.18 ^B,a^ ± 0.11	5.88 ^E,b^ ± 0.02	4.63 ^E,F,c^ ± 0.02
**29**	5.35 ^E,F,a^ ± 0.07	5.21 ^E,F,b^ ± 0.01	4.19 ^G,H,c^ ± 0.02	5.69 ^C,a^ ± 0.01	5.61 ^F,b^ ± 0.01	4.61 ^E,F,c^ ± 0.00
**35**	5.33 ^F,a^ ± 0.06	5.17 ^F,b^ ± 0.01	4.19 ^F,G,H,c^ ± 0.03	5.55 ^D,a^ ± 0.04	5.40 ^G,a^ ± 0.06	4.57 ^F,G,b^ ± 0.04
**41**	5.34 ^E,F,a^ ± 0.01	5.04 ^G,b^ ± 0.04	4.24 ^E,F,c^ ± 0.00	5.31 ^E,a^ ± 0.01	5.29 ^H,a^ ± 0.01	4.58 ^F,G,b^ ± 0.02
**47**	5.22 ^F,G,a^ ± 0.01	5.02 ^G,b^ ± 0.01	4.24 ^E,F,G,c^ ± 0.01	5.24 ^E,a^ ± 0.04	5.12 ^I,b^ ± 0.01	4.54 ^G,c^ ± 0.01

^A,B,C,D,E,F,G,H,I^ Means with different uppercase letters within the same treatment are significantly different (*p* < 0.05). ^a,b,c^ Means with different lowercase letters within the same storage day are significantly different (*p* < 0.05).

**Table 3:** Changes in pH values for ham slices treated or not with HPP, during storage at 12 °C.

pH Values at 12 °C						
Days	Without HPP Treatment			With HPP Treatment		
	Control	PF Film	PS Film	Control	PF Film	PS Film
**0**	6.49 ^A,a^ ± 0.07	6.49 ^A,a^ ± 0.07	6.49 ^A,a^ ± 0.07	6.54 ^A,a^ ± 0.11	6.54 ^A,a^ ± 0.11	6.54 ^A,a^ ± 0.11
**3**	6.01 ^B,a^ ± 0.01	5.93 ^B,b^ ± 0.03	4.75 ^B,c^ ± 0.03	6.43 ^A,a^ ± 0.06	6.19 ^B,b^ ± 0.02	5.28 ^B,c^ ± 0.04
**6**	5.62 ^C,a^ ± 0.01	5.30 ^C,b^ ± 0.08	4.19 ^C,D,c^ ± 0.02	6.42 ^A,a^ ± 0.01	6.10 ^C,b^ ± 0.03	4.50 ^C,D,c^ ± 0.00
**10**	5.46 ^D,a^ ± 0.01	4.92 ^E,b^ ± 0.07	4.06 ^E,c^ ± 0.01	6.15 ^B,a^ ± 0.02	6.03 ^D,b^ ± 0.01	4.42 ^E,c^ ± 0.04
**13**	5.42 ^D,a^ ± 0.01	5.17 ^C,D,b^ ± 0.00	4.08 ^E,c^ ± 0.04	5.76 ^C,a^ ± 0.05	5.60 ^E,b^ ± 0.02	4.50 ^C,D,c^ ± 0.02
**17**	5.35 ^D,a^ ± 0.01	4.96 ^E,b^ ± 0.11	4.12 ^D,E,c^ ± 0.01	5.54 ^D,a^ ± 0.02	5.49 ^F,a^ ± 0.01	4.44 ^D,E,b^ ± 0.02
**20**	5.45 ^D,a^ ± 0.04	5.02 ^D,E,b^ ± 0.13	4.13 ^D,E,c^ ± 0.04	5.25 ^E,a^ ± 0.06	5.09 ^G,b^ ± 0.01	4.53 ^C,c^ ± 0.04
**24**	5.24 ^E,a^ ± 0.02	5.07 ^D,E,a^ ± 0.04	4.26 ^C,b^ ± 0.08	5.16 ^F,a^ ± 0.01	5.03 ^G,b^ ± 0.04	4.53 ^C,c^ ± 0.04
**27**	5.23 ^E,a^ ± 0.12	5.02 ^D,E,a^ ± 0.03	4.09 ^E,b^ ± 0.01	5.12 ^F,G,a^ ± 0.05	4.87 ^H,b^ ± 0.07	4.40 ^E,c^ ± 0.03
**33**	5.06 ^F,a^ ± 0.05	5.00 ^E,a^ ± 0.01	4.08 ^E,b^ ± 0.03	5.06 ^G,H,a^ ± 0.04	4.91 ^H,b^ ± 0.05	4.46 ^D,E,c^ ± 0.03
**40**	5.04 ^F,a^ ± 0.04	4.95 ^E,a^ ± 0.06	4.13 ^D,E,b^ ± 0.03	4.98 ^H,a^ ± 0.01	4.87 ^H,b^ ± 0.02	4.51 ^C,c^ ± 0.01

^A,B,C,D,E,F,G,H^ Means with different uppercase letters within the same treatment are significantly different (*p* < 0.05). ^a,b,c^ Means with different lowercase letters within the same storage day are significantly different (*p* < 0.05).

**Table 4:** Changes in C* values for ham slices treated or not with HPP, during storage at 4 °C.

Chroma C* at 4 °C						
Days	Without HPP Treatment			With HPP Treatment		
	Control	PF Film	PS Film	Control	PF Film	PS Film
**0**	26.99 ^A,a^ ± 2.48	26.99 ^A,a^ ± 2.48	26.99 ^A,a^ ± 2.48	25.05 ^A,a^ ± 0.87	25.05 ^A,a^ ± 0.87	25.05 ^A,a^ ± 0.87
**6**	25.59 ^A,a^ ± 0.74	26.16 ^A,a^ ± 0.65	27.90 ^A,a^ ± 2.62	24.97 ^A,a^ ± 0.47	25.54 ^A,a^ ± 0.73	26.45 ^A,a^ ± 1.24
**12**	25.67 ^A,a^ ± 0.32	26.62 ^A,a^ ± 0.04	28.33 ^A,a^ ± 1.71	25.64 ^A,B,a^ ± 0.27	26.17 ^A,a^ ± 0.68	26.06 ^A,a^ ± 1.58
**18**	26.04 ^A,a^ ± 1.08	26.40 ^A,a^ ± 0.06	28.48 ^A,a^ ± 2.51	26.52 ^B,a^ ± 1.06	25.94 ^A,a^ ± 0.53	26.67 ^A,a^ ± 0.78
**24**	24.63 ^A,a^ ± 0.66	26.25 ^A,a^ ± 0.12	27.42 ^A,a^ ± 3.12	25.12 ^A,B,a^ ± 0.65	25.79 ^A,a^ ± 0.32	27.96 ^A,a^ ± 2.71
**31**	25.98 ^A,a^ ± 0.15	26.33 ^A,a^ ± 3.58	25.12 ^A,a^ ± 0.45	24.75 ^A,a^ ± 0.05	28.11 ^B,a^ ± 1.28	26.65 ^A,a^ ± 3.67
**38**	24.94 ^A,a^ ± 1.58	26.08 ^A,a^ ± 1.04	27.53 ^A,a^ ± 2.94	25.66 ^A,B,a^ ± 0.72	26.58 ^A,B,a^ ± 0.60	27.34 ^A,a^ ± 3.88
**45**	25.45 ^A,a^ ± 0.87	25.40 ^A,a^ ± 0.58	27.52 ^A,a^ ± 3.63	25.44 ^A,B,a^ ± 0.96	26.40 ^A,a^ ± 0.42	28.43 ^A,a^ ± 3.73
**52**	25.20 ^A,a^ ± 0.29	25.68 ^A,a^ ± 1.13	26.77 ^A,a^ ± 3.00	25.33 ^A,B,a^ ± 0.62	24.91 ^A,a^ ± 0.23	27.44 ^A,a^ ± 3.69
**59**	25.37 ^A,a^ ± 0.99	26.11 ^A,a^ ± 0.43	26.55 ^A,a^ ± 2.82	25.42 ^A,B,a^ ± 0.21	26.49 ^A,B,a^ ± 0.85	26.70 ^A,a^ ± 3.59
**66**	26.48 ^A,a^ ± 0.42	26.09 ^A,a^ ± 0.24	26.13 ^A,a^ ± 3.37	25.72 ^A,B,a^ ± 0.36	25.46 ^A,a^ ± 0.83	27.37 ^A,a^ ± 3.71

^A,B^ Means with different uppercase letters within the same treatment are significantly different (*p* < 0.05). ^a^ Means with different lowercase letters within the same storage day are significantly different (*p* < 0.05).

**Table 5:** Changes in C* values for ham slices treated or not with HPP, during storage at 8 °C.

Chroma C* at 8 °C						
Days	Without HPP Treatment			With HPP Treatment		
	Control	PF Film	PS Film	Control	PF Film	PS Film
**0**	26.99 ^A,a^ ± 2.48	26.99 ^A,a^ ± 2.48	26.99 ^A,a^ ± 2.48	25.05 ^A,a^ ± 0.87	25.05 ^A,a^ ± 0.87	25.05 ^A,a^ ± 0.87
**4**	25.42 ^A,a^ ± 0.06	26.04 ^A,a^ ± 0.55	27.81 ^A,a^ ± 1.93	25.06 ^A,a^ ± 0.09	28.38 ^A,a^ ± 3.25	28.17 ^A,a^ ± 2.42
**8**	26.34 ^A,a^ ± 1.00	25.95 ^A,a^ ± 0.81	28.38 ^A,a^ ± 2.22	25.68 ^A,a^ ± 0.22	26.46 ^A,a^ ± 0.46	28.62 ^A,a^ ± 1.78
**12**	24.95 ^A,a^ ± 0.15	26.19 ^A,a^ ± 0.31	28.25 ^A,a^ ± 2.41	25.24 ^A,a^ ± 0.02	25.99 ^A,a^ ± 0.77	28.05 ^A,a^ ± 3.70
**17**	25.42 ^A,a^ ± 0.77	26.18 ^A,a^ ± 0.31	27.17 ^A,a^ ± 2.98	25.35 ^A,a^ ± 0.90	26.42 ^A,a^ ± 1.11	27.26 ^A,a^ ± 3.31
**21**	25.19 ^A,a^ ± 0.85	25.96 ^A,a^ ± 0.34	27.59 ^A,a^ ± 2.92	26.02 ^A,a^ ± 1.05	28.27 ^A,a^ ± 3.10	24.63 ^A,a^ ± 0.14
**25**	24.91 ^A,a^ ± 0.17	25.92 ^A,a^ ± 0.32	26.53 ^A,a^ ± 4.29	25.42 ^A,a^ ± 1.00	25.94 ^A,a^ ± 0.61	28.11 ^A,a^ ± 2.62
**29**	25.86 ^A,a^ ± 1.01	25.84 ^A,a^ ± 0.31	27.05 ^A,a^ ± 3.47	25.09 ^A,a^ ± 0.71	27.65 ^A,a^ ± 2.65	24.57 ^A,a^ ± 0.47
**35**	24.88 ^A,a^ ± 1.57	25.52 ^A,a^ ± 0.24	27.46 ^A,a^ ± 2.14	24.88 ^A,a^ ± 0.08	25.90 ^A,a^ ± 0.10	24.43 ^A,a^ ± 0.24
**41**	24.85 ^A,a^ ± 1.60	24.86 ^A,a^ ± 1.46	27.48 ^A,a^ ± 2.72	25.31 ^A,a^ ± 0.59	25.76 ^A,a^ ± 0.64	24.75 ^A,a^ ± 1.29
**47**	25.67 ^A,a^ ± 1.50	25.31 ^A,a^ ± 0.05	27.83 ^A,a^ ± 1.13	25.87 ^A,a^ ± 0.38	25.22 ^A,a^ ± 0.58	23.66 ^A,a^ ± 0.15

^A^ Means within the same treatment are not significantly different (*p* > 0.05). ^a^ Means within the same storage day are not significantly different (*p* > 0.05).

**Table 6:** Changes in C* values for ham slices treated or not with HPP, during storage at 12 °C.

Chroma C* at 12 °C						
Days	Without HPP Treatment			With HPP Treatment		
	Control	PF Film	PS Film	Control	PF Film	PS Film
**0**	26.99 ^A,a^ ± 2.48	26.99 ^A,a^ ± 2.48	26.99 ^A,a^ ± 2.48	25.05 ^A,B,a^ ± 0.87	25.05 ^A,B,a^ ± 0.87	25.05 ^A,a^ ± 0.87
**3**	24.69 ^A,a^ ± 0.40	26.45 ^A,a^ ± 3.09	26.74 ^A,a^ ± 1.15	25.06 ^A,B,a^ ± 0.22	25.32 ^A,B,a^ ± 1.55	25.06 ^A,a^ ± 1.34
**6**	24.83 ^A,a^ ± 0.81	27.76 ^A,a^ ± 2.51	27.95 ^A,a^ ± 3.00	25.49 ^A,B,C,a^ ± 0.42	25.81 ^A,B,a^ ± 1.22	26.60 ^A,a^ ± 3.61
**10**	25.26 ^A,a^ ± 0.99	26.38 ^A,a^ ± 1.23	27.83 ^A,a^ ± 2.65	25.44 ^A,B,C,a^ ± 0.12	26.44 ^A,B,a^ ± 1.32	26.83 ^A,a^ ± 2.87
**13**	25.23 ^A,a^ ± 1.03	25.77 ^A,a^ ± 0.22	27.60 ^A,a^ ± 2.40	25.30 ^A,B,C,a^ ± 0.34	26.55 ^A,B,a^ ± 0.39	24.34 ^A,a^ ± 0.40
**17**	24.74 ^A,a^ ± 0.81	25.78 ^A,a^ ± 0.96	27.01 ^A,a^ ± 3.38	25.86 ^B,C,a^ ± 0.10	26.37 ^A,B,a^ ± 0.35	26.52 ^A,a^ ± 3.12
**20**	25.51 ^A,a^ ± 1.61	25.55 ^A,a^ ± 0.65	27.29 ^A,a^ ± 1.39	24.66 ^A,B,a^ ± 1.15	25.86 ^A,B,a^ ± 0.29	26.39 ^A,a^ ± 3.77
**24**	25.51 ^A,a^ ± 1.06	25.84 ^A,a^ ± 0.65	26.64 ^A,a^ ± 3.07	25.80 ^A,B,C,a^ ± 0.62	25.72 ^A,B,a^ ± 0.05	27.26 ^A,a^ ± 4.17
**27**	25.53 ^A,a^ ± 0.74	24.80 ^A,a^ ± 1.69	27.80 ^A,a^ ± 2.69	26.47 ^C,a^ ± 0.03	26.07 ^A,B,a^ ± 1.09	27.65 ^A,a^ ± 3.72
**33**	24.14 ^A,a^ ± 0.67	25.66 ^A,a^ ± 0.35	27.36 ^A,a^ ± 1.99	24.55 ^A,a^ ± 0.42	27.60 ^B,a^ ± 2.49	26.43 ^A,a^ ± 3.17
**40**	23.75 ^A,a^ ± 1.22	25.86 ^A,a^ ± 1.17	26.31 ^A,a^ ± 2.83	24.53 ^A,a^ ± 0.62	24.04 ^A,a^ ± 0.22	28.06 ^A,a^ ± 2.87

^A,B,C^ Means with different uppercase letters within the same treatment are significantly different (*p* < 0.05). ^a^ Means within the same storage day are not significantly different (*p* > 0.05).
